# Frameworks for embedding a research culture in allied health practice: a rapid review

**DOI:** 10.1186/s12961-018-0304-2

**Published:** 2018-03-21

**Authors:** Susan C. Slade, Kathleen Philip, Meg E. Morris

**Affiliations:** 1 La Trobe Centre for Sport and Exercise Medicine Research, School of Allied Health, College of Science, Health & Engineering, La Trobe University, Bundoora, 3086 Australia; 2 Department of Health and Human Services, Victoria State Government, 50 Lonsdale Street, Melbourne, Vic 3000 Australia; 3 Healthscope, North Eastern Rehabilitation Centre, Ivanhoe, Australia

**Keywords:** research capacity, allied health, policy, systematic review knowledge translation, implementation science, leadership

## Abstract

**Background:**

Although allied health clinicians play a key role in the provision of healthcare, embedding a culture of research within public and private health systems remains a challenge. In this rapid review we critically evaluate frameworks for embedding research into routine allied health practice, as the basis for high quality, safe, efficient and consumer-focused care.

**Methods:**

A rapid review (PROSPERO: CRD42017075699) was conducted to evaluate frameworks designed to create and embed research in the health sector. Included were full-text, English-language, peer-reviewed publications or Government reports of frameworks that could inform the implementation of an allied health research framework. Eight electronic databases and four government websites were searched, using search terms such as models, frameworks and research capacity-building. Two independent researchers conducted all review stages and used content and thematic analysis to interpret the results.

**Results:**

Sixteen framework papers were finally included. Content analysis identified 44 system and regulatory level items that informed the research frameworks, 125 healthcare organisation items and 76 items relating to individual clinicians. Thematic analysis identified four major themes. Firstly, sustainable change requires allied health research policies, regulation, governance and organisational structures that support and value evidence-based practice. Secondly, research capability, receptivity, advocacy and literacy of healthcare leaders and managers are key to successful research implementation. Third, organisational factors that facilitate a research culture include dedicated staff research positions, time allocated to research, mentoring, professional education and research infrastructure. When healthcare agencies had strong partnerships with universities and co-located research leaders, research implementation was strengthened. Finally, individual attributes of clinicians, such as their research skills and capabilities, motivation, and participation in research teams, are essential to embedding research into practice.

**Conclusion:**

Theoretical frameworks were identified that informed processes to embed a culture of allied health research into healthcare services. Research-led and evidence-informed allied health practice enables optimisation of workforce capability and high-quality care.

## Background

Allied health services are a major pillar of health and social care worldwide and allied health professionals constitute approximately one-third of the health workforce [1]. The term ‘allied health’ refers to a broad range of health disciplines, excluding doctors, nurses and midwives, dentists, and complementary therapists. Allied health can include disciplines such as physiotherapy, speech therapy, occupational therapy, podiatry, psychology, dietetics, pharmacy, prosthetics, orthotics, orthoptics, radiology, medical science, social work and exercise physiology [2], although this varies across the globe.

Allied health clinicians play a key role in promoting health and wellbeing in the public and private healthcare sectors [3]. As well as managing impairments, disabilities and participation restrictions [4], allied health professionals bridge the gap between the medical and nursing professions, advocate for patients and their families, and foster inter-professional teams and multi-disciplinary care [1, 2, 4–8]. Allied health professionals are encouraged to be research literate [6], and to assist the translation of research evidence into clinical practice to optimise patient outcomes [9]. Some are also research generators [10] and others focus on research implementation [11] to bridge the evidence-practice gap [12].

Evidence-based practice is central to effective, efficient, consumer-focused healthcare. It centres around the principles of (1) best available evidence, (2) clinical expertise and (3) incorporating consumer preferences into practice [13]. Despite clinical expertise and a quality focus, some allied health professionals lack research and evaluation skills [14–16]. Clinical practice has traditionally been directed towards patient care and resource allocation, with allied health clinicians being ‘consumers’ of research [6]. This is evolving, and more allied health professionals are now becoming involved in research training, knowledge generation, knowledge translation, evidence implementation, policy setting, research partnerships, co-production and research leadership [1, 2, 7, 10, 17, 18].

Underpinning evidence-based practice is a strong research culture with a framework that enables service planning, decision-making and sustained integration of evidence-based healthcare [19–21]. Governments have increasingly recognised that resources are optimised and health outcomes are improved when health policy and programme design are informed by evidence from research [22–24]. A functioning research culture is necessary to enable this research generation [9]. There is a need for research capacity-building in allied health to develop individuals to higher levels of skill, which will enable them to conduct quality research and translate the findings to improve patient outcomes.

There also exists a need to improve the ability of individuals, organisations and systems to conduct, use and promote research through providing training, funding, infrastructure, linkages and career pathways [18, 25–27]. Some of the main reasons for building research capacity and a research culture are to adopt evidence-based practice, generate new knowledge, achieve research objectives, strengthen workforce research literacy and assist workforce recruitment, retention and job satisfaction [12, 18, 20, 28, 29].

A strategic approach to research capacity-building is needed to accommodate the complex and multi-disciplinary context of allied healthcare [1, 8, 30]. The strategies that have been traditionally used to build research capability and capacity have mainly focused on processes, such as skill development, in evidence-based practice, journal clubs or quality projects [1, 2, 6, 19, 31, 32]. They have also focused on research training, such as grant writing, conference presentations, publication writing, and encouragement to participate in research networks and partnerships [5, 28, 33–40]. Despite the need, there is no current framework for embedding an allied health research culture across allied health practice in public or private healthcare systems.

As the basis for developing a future policy framework to embed allied health research into routine clinical practice, this review shall critically evaluate the published worldwide literature on theories and frameworks that have been designed and developed to create and embed research capacity in the allied health clinical sector. A framework provides (1) the lens through which research capacity-building strategies are developed and evaluated; (2) the potential determinants and domains of research implementation (including individual, organisational and policy factors); (3) research engagement actions; and (4) mechanisms for research to inform policy and practice [22, 26].

Of particular interest in this review were frameworks to build research capability, capacity and implementation. We also searched for frameworks that incorporated a broader systems level and policy viewpoint so that research implementation did not solely rest in the hands of individual clinicians. We propose that allied health clinical practice can be enhanced by embedding a research culture into routine service provision within the clinical environment. The implementation of policies, systems, environments and leadership models empowering clinicians to incorporate research as a routine part of their role were also foci of this rapid review.

### Research question

What allied health research frameworks and models have robust evidence to enable a research culture to be embedded into routine allied health clinical practice?

### Aims

This review shall inform the future design of an allied health framework to foster a culture of research in allied health practice. As a first step, the primary aims of this rapid review are to (1) identify existing research capacity-building and capability-building and research culture frameworks/models, as well as to (2) synthesise existing evidence to identify the essential elements for embedding a culture of research within allied health practice. The secondary aim is to summarise the strengths and limitations of existing frameworks and models.

## Methods

This rapid review was commissioned by the Department of Health and Human Services, Victoria, Australia, and was registered with the international prospective register of systematic reviews (PROSPERO: CRD42017075699). The rapid review was conducted and informed by Cochrane guidelines [41] and rapid review methods [42, 43], and reported according to the Preferred Reporting Items for Systematic Reviews and Meta-Analyses (PRISMA) Statement [44] and the Enhancing Transparency in Reporting the synthesis of Qualitative research [45]. The a priori inclusion and exclusion criteria were established before conducting searches of the electronic databases and were applied to the final search yield. All review stages were conducted by two independent reviewers, who collaborated when necessary to reach consensus.

### Eligibility criteria

Eligibility criteria were established before searching electronic databases. Papers were included if they were full text English-language, published in peer-reviewed journals or on organisation/Government websites, reported frameworks, models for building research capacity and culture in healthcare, and provided items for review and/or evaluation. Broad healthcare models could be included for later evaluation of applicability to allied health. Editorials and opinion pieces were excluded.

### Definitions

1. A ‘Theory’ was defined as “*a system of ideas that provide an explanation and/or a set of principles on which the practice of an activity is based*” [46]. It has also been defined as “*a set of analytical principles or statements designed to structure our observation, understanding and explanation of the world*” [47].
2. A ‘Model’ was defined as “*a deliberate simplification of a phenomenon or a specific aspect of a phenomenon*”, “*closely related to theory*” and “*a model is descriptive while a theory is explanatory*” [47].
3. A ‘Frameworks’ was defined as “*a structure, overview, outline, system or plan consisting of various descriptive categories, e.g. concepts, constructs or variables, and the relations between them that are presumed to account for a phenomenon*” [47]. In this review, we considered that a framework would inform polices, decisions and judgments about evidence-based allied health practice. The framework could also specify potential individual, system and organisational determinants of research use, research engagement and knowledge dissemination [22].
4. ‘Research capacity-building’ was defined as the “*process of individual and institutional development which leads to higher levels of skills and greater ability to perform useful research*” [26]. Key common elements across research capacity-building include shared goals, collaboration and partnership, education and training, organisation support and leadership, evaluation, and monitoring. It may be considered as a continuum of clinician research development from a research consumer to a research active clinician and then to a research leader. It is an approach to the development of sustainable skills, organisational structures, resources and commitments to improvement in health and other sectors to multiply health gains [26].

### Identification of included papers

Electronic databases were searched without date limits up until October 15, 2017, using explosions and combinations of key search terms such as allied healthcare, allied health clinicians, allied health, framework, model, theory, research capacity, capacity-building, research capacity-building, research culture, clinical research, research culture, organisational role, motivation theory, health researcher, framework, theory, model, policy, allied health, translation, implementation, leadership, and governance***.*** A sample MEDLINE search strategy is included in Appendix 1.

The following eight databases were searched: CINAHL, Embase, MEDLINE, PubMed, PsychInfo, Health and Psychological Instruments, Global Health and Google Scholar. Websites included the Government of Canada Publications, United Kingdom Department of Health, Victorian Government Library Services catalogue, the Primary Health Care Research and Information Service, and Australian Government health websites. Reference lists of relevant reports were searched, and other relevant work sought through citation tracking, the grey literature, consultation with the Research and Liaison Librarian at the Victorian Government Library Service and contact with content and research experts. All of the searches were downloaded to a reference database for deletion of duplicates and initial screening of titles by the primary author, who deleted those that were clearly irrelevant.

Two reviewers independently reviewed the search results, deleted duplicates, and screened titles and abstracts for exclusion of reports that did not meet the eligibility criteria. Full text copies were obtained for all potentially relevant reports, independently screened against the eligibility criteria and read in full before final inclusion/exclusion.

### Method quality appraisal

Method quality appraisal was conducted when there was a validated instrument for the appropriate empirical study design such as the preliminary Mixed Methods Appraisal Tool (MMAT) [48] for mixed methods studies or the Critical Appraisal Skills Programme (CASP) Checklist [49] for qualitative empirical studies. In the absence of a validated instrument we described the reported psychometric properties or provided a narrative summary of elements that may have contributed to the risk of bias. We used guidance from the National Collaborating Centre for Methods and Tools [50].

### Data extraction

Data extraction guidelines were developed so that the same information was extracted from each included paper and systematically extracted into spreadsheets under the pre-specified headings of first author, title, year, journal or organisation or publisher, domain, theory, model, items, and the conceptual framework. Items were extracted independently by two researchers from each framework or from the text of the report. The research capacity-building titles were identified and their items were assembled into groups that addressed common targets. Common targets included headings that incorporated words such as individual, organisation, system or policy. Under these headings we included items such as enablers, barriers, skills, self-efficacy, policies and procedures, management, legislation, regulation, etc. The completed data extraction forms were examined for consistency. Following discussion, these were merged for the data synthesis phase.

### Data synthesis

Two independent reviewers (SS, MM) conducted thematic and content analysis and consulted during the process using the constant comparison method, which (1) summarising and synthesising the item content with data reduction of items into ‘like’ categories and (2) the formation of themes through overarching similarities and connections. No prior theory was used to assist in identification of items and frameworks, but rather iterative rounds of open data-driven inductive coding were used [51–53]. The content analysis aimed to identify the key items that were important for embedding a research culture. The thematic analysis aimed to build an understanding of the broader framework of research capacity-building.

## Results

The total search yield of 1255 articles was sorted by title and 1158 clearly unsuitable titles were excluded. Five papers were added from reference lists and the remaining 97 titles were examined by title and abstract. Sixty-eight papers were excluded after applying the eligibility criteria to the information contained in the abstract. Of the remaining 34 papers, two reviewers independently, and by consensus, excluded 23 papers after reading them in detail and applying the eligibility criteria.

**Fig. 1:**
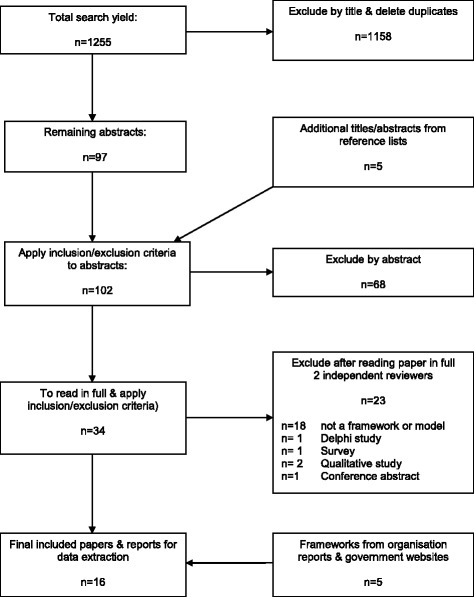
PRISMA-compliant flowchart for inclusion into the review

Figure 1 shows a flowchart of progress into the review, indicating the papers/documents that met the criteria and were included for data extraction, as well as the final exclusions. The final 16 included papers and organisation/government reports contained 16 discrete research capacity-building frameworks and models that included domains and items for data extraction and synthesis (Table 1) [19, 22–28, 30, 54–60]. Twenty-three papers were excluded in the final round; 18 did not report a framework or model, one was a conference abstract, and four were qualitative or investigative studies (Appendix 2). Frameworks for research capacity-building in healthcare had been developed and implemented in Australia, Canada, United Kingdom and United States of America and date from 2001 to 2017. The extracted frameworks are summarised in Table 1.

**Table 1:** Included papers

Author,Year	Title	Framework or Model	Study location/Healthcare domain	Participants	Framework development/Study type
Brennan, 2017 [54]	Development and validation of SEER (Seeking, Engaging with and Evaluating Research): a measure of policymakers’ capacity to engage with and use research	SEER Framework	Australia, Health policy-makers	Investigator team (researchers, policy-makers) 150/272 respondents, 57/105 respondents, 9 policy agencies	Item generation and refinement, Literature review and expert consensus survey, Validity and internal consistency survey, Test-retest reliability
Cooke, 2005 [26]	A framework to evaluate research capacity building in health care	Cooke Framework	United Kingdom, Primary care	Not reported	Literature review and expert opinion
Farmer, 2002 [19]	A conceptual model for capacity building in Australian primary health care research	‘Whole system’ Framework	Australia, General practitioners	Not reported	Expert opinion
Fleisher, 2007 [55]	The NCI’s Cancer Information Service’s Research Continuum Framework: integrating research into cancer education practice	CIS Research Continuum Framework	United States, National Cancer Information Service	Not reported	Not reported
Golenko, 2012 [25]	A thematic analysis of the role of the organisation in building allied health research capacity: a senior managers’ perspective	Research Capacity-Building Model	Australia, Allied health managers	Nine semi-structured interviews	Qualitative study with thematic analysis
Gullick, 2016 [56]	Building research capacity and productivity among advanced practice nurses: an evaluation of the Community of Practice model	Wenger’s Community of Practice Model	Australia, Nursing	Six focus groups (25 participants: 2 nurse practitioners; 23 clinical nurse consultants)	Qualitative study with thematic analysis
Holden, 2012 [27]	Validation of the research capacity and culture (RCC) tool: measuring RCC at individual, team and organisation levels	Research Capacity and Culture Tool	Australia, Primary care	Allied health assistants = 3; Dieticians = 10; Occupational therapists = 24; Physiotherapists = 29; Speech pathologists = 10; Social workers = 20; Psychologists = 6; Doctors, nurses = 14	Quantitative methods with factor analysis, test-retest reliability, intra-class correlation
Hulcombe, 2014 [28]	An approach to building research capacity for health practitioners in a public health environment: an organisational perspective	Research Capacity and Culture Building Framework	Australia, Allied health clinicians	Medical laboratory assistants; Nutrition and dietetics; Occupational therapy; Oral health therapists; Physiotherapy; Podiatry; Psychology; Public health practitioners; Radiation therapy	Literature review, stakeholder consultations, expert opinion; Development of health practitioners (Queensland Health) certified agreement (No. 2) (HPEB2) – CA/2011/106
McCance, 2006 [57]	Developing a best practice framework to benchmark research and development activity in nursing and midwifery	Research and Development Best Practice Framework	United Kingdom, Nursing	Not reported	Literature review that included 52 papers and generated six best practice statements
Makkar, 2016 [58]	The development of ORACLe: a measure of an organisation’s capacity to engage in evidence-informed health policy	ORACLe Framework	Australia, Health policy-makers	Nine semi-structured interviews – item content; Six semi-structured interviews – item wording	Literature review to generate items; Qualitative methods with content analysis for key domains; Quantitative methods to develop a scoring system and psychometric testing (*n* = 24)
Makkar, 2016 [59]	The development of SAGE: A tool to evaluate how policymakers’ engage with and use research in health policymaking	SAGE Framework	Australia, Health policy-makers	65 interviews with policy-makers	Literature review and expert consultation to develop item content and wording; Qualitative methods but not reported; Quantitative methods to develop a scoring system and psychometric testing
Redman, 2015 [22]	The SPIRIT Action Framework: A structured approach to selecting and testing strategies to increase the use of research in policy	SPIRIT Action Framework	Australia, Health policy-makers	Nine semi-structured interviews with policy-makers – item content	Literature review including 106 papers from which items were generated; Qualitative methods with content analysis and a review of framework domains; Expert opinion
Ried, 2006 [60]	Setting directions for capacity building in primary health care: a survey of a research network	SARNet Framework	Australia, Primary healthcare	Allied health = 26General practitioners = 19Health services = 11Nurses = 9Academics = 9Hospital doctors = 7	Qualitative and quantitative methods with unreported design and methods
Whitworth, 2012 [30]	Enhancing research capacity across healthcare and higher education sectors: development and evaluation of an integrated model	Partnership Model	United Kingdom, Speech therapists	Speech and language therapists	Expert opinion from senior managers; Research ideas were solicited from practitioners; Qualitative methods to explore experiences of the research collaboration
NSW Health, 2001 [23]	A Framework for Building Capacity to Improve Health	RCB Framework	Australia, Primary healthcare	Not reported	Expert opinion
Hotte, 2015 [24]	Building Research Capacity within the British Columbia Health Authorities: health services and policy research support network	Health Authority Capacity-Building Program	Canada, Public health	Not reported	Literature review and identification of six themes

### Method quality appraisal

Due to a lack of reported data, we were unable to apply the MMAT and CASP checklists to 10 of the included frameworks. Five of these frameworks were derived by expert opinion and evidence synthesis and will require validity and reliability testing [19, 26, 30, 55, 57]. Three frameworks were derived from literature reviews and unreported qualitative/quantitative methods [28, 56, 60]. Two frameworks were government reports and guidance documents derived from expert opinion and required implementation and effectiveness testing [23, 24].

The SPIRIT Action Framework was developed by literature synthesis, interviews with policy-makers and an iterative process of pragmatic tool development. The SPIRIT is not specific to allied health and requires implementation and effectiveness testing for allied health professions. It can guide conceptually informed practical decisions in the selection and testing of interventions to increase the use of research in policy. It scored 100% on the MMAT Appraisal Tool for quality of qualitative and mixed methods [22].

The Thematic Model for Research Capacity Building was informed by qualitative research methods using structured interviews that were thematically analysed. Four key themes formed the foundation of a research capacity-building framework. It scored 100% on the MMAT for the quality of the qualitative methods and satisfied all components of the CASP [25].

The Wenger’s Community of Practice Model was informed by qualitative research methods using focus groups that were thematically analysed. It scored 100% on the MMAT for the quality of the qualitative methods and satisfied all components of the CASP [26].

The SEER Framework was informed by literature synthesis, item generation and refinement, consultation with policy-makers, and testing of measurement properties. It demonstrated good internal consistency and reliability but was not specific to allied health. The four included scales may be used in policy settings to evaluate current capacity and identify areas that need capacity-building. It scored 100% on the MMAT Appraisal Tool for quality of mixed methods [54].

The Research Capacity and Culture tool was developed by a literature review and evidence synthesis. Psychometric testing in a Queensland primary healthcare sample (*n* = 134) demonstrated excellent internal consistency for organisation, team and individual domains, and strong test-retest reliability. The Research Capacity and Culture tool was not specifically designed for allied health alone and requires translation and effective testing. It scored 100% on the MMAT Appraisal Tool for quality of descriptive quantitative methods [27].

The ORACLE Framework was derived by robust mixed methods that included qualitative methods for face validity and quantitative methods for scoring a matrix. It was designed to score the capacity of an organisation to use research in policy-making but is not specific to allied health. It has yet to be validated as a measure of organisational capacity and culture to support research use. It scored 100% on the MMAT Appraisal Tool for quality of qualitative, descriptive quantitative and mixed methods [58].

The SAGE Framework was derived by interviews with policy-makers and document analysis but the explicit qualitative methods were not described. It was designed to measure the extent to which research was engaged with and used in a discrete policy or programme document. It was not designed to identify overarching organisational structures that may contribute to barriers to research use and psychometric testing for validity and reliability is planned. It scored 100% on the MMAT Appraisal Tool for quality of descriptive quantitative and mixed methods [59].

### Content analysis

**Fig. 2:**
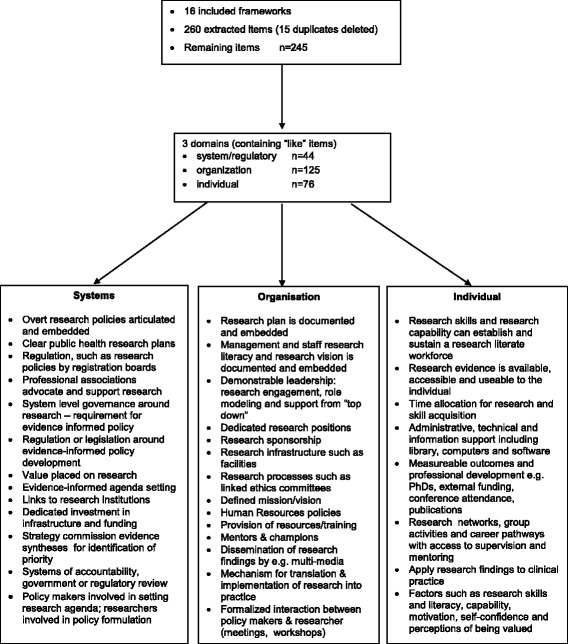
Content analysis – item reduction of identified research capacity-building frameworks and models

A total of 260 items were extracted from the 16 frameworks and 15 duplicate items were deleted. From the remaining framework items, and from our data analysis and interpretation of these data, we identified the following three domains by consensus: (1) system or regulatory (44 items), (2) organisation (125 items) and (3) individual (76 items). Through an iterative review process and constant comparison, the items with similar content and meaning were grouped using the domain headings summarised in Fig. 2.

### Thematic analysis

The main over-arching theme identified in this review was that provision of research-informed healthcare that is consistent with best available evidence requires over-arching high level policies to enable leadership, organisations and individuals to embed a research culture into everyday allied health practice. Four key themes were identified, as shown in Table 2.

**Table 2:** Themes identified in the data analysis

Theme	Title and summary
Overarching theme	The provision of research-informed healthcare that is consistent with best available evidence requires over-arching policies that enable the organisation and individuals to be research active
Theme 1	Regulatory environment, governance and organisational structures• Sustainable change requires allied health research policies, regulation, governance and organisational structures that support and value evidence-based practice
Theme 2	Leadership and management buy-in• Research capability, receptivity and literacy of healthcare leaders and managers are key to successful research implementation
Theme 3	Systems, tools, resources and time• The provision of research infrastructure, research systems, tools, databases, resources, time allocation, dedicated research staff positions, mentoring, professional education and mechanisms for recognition and reward are key organisational factors that enable research capacity-building• Partnerships between healthcare agencies and universities with co-located research leaders optimises research quality and productivity
Theme 4	Attributes of individual clinicians• Attributes and capabilities of individual clinicians such as research qualifications, skills, research literacy, communication skills, partnerships, confidence and motivation help strengthen and develop research interactions and increase research receptivity

### Theme 1: Regulatory environment, governance and organisational structures

All of the included frameworks provided descriptions of over-riding policies, governance frameworks and regulatory systems considered as essential for sustaining a culture of scientific enquiry and evidence-based practice [15, 18–24, 26, 50–56]. These were applicable across the entire allied healthcare domain, as well as for medicine and nursing. Sustainable change was argued to require an environment that supports and values the development, and continuation, of research and evaluation processes [19, 22, 25, 27, 57–59].

Evidence-informed policy-making needs to be understood and implemented, particularly the incentives for policy-makers to support the use of evidence in policy cycles [11, 28]. Strengthening the appreciation and capacity of individual policy-makers, and their organisations, to make greater use of evidence can be a first step in generating better evidence-informed policy. Policy-makers can be informed about, and benefit from, evidence-informed policy and can also be assisted by tools to help them to access, analyse and utilise evidence. They can be encouraged to engage more closely with researchers as policy advisors. Collaborations with, and skills acquisition by, policy-makers were reported as important factors that influence the use of research results and evidence [19, 22–27, 30, 54–60].

### Theme 2: Leadership and management buy-in

Common to all frameworks were themes of research as the ‘core business’ with strong leadership and investment, by management, in evidence-informed policy and the acquisition of research literacy [19, 22–27, 30, 55–60]. There was also consensus that individual allied health clinicians could further benefit from active and deliberate support to enable them to progress from being a non-participant in research to becoming truly research active and evidence informed [23, 24, 30, 58–60]. Strong recommendations were made to embed formal engagement and collaboration with researchers and research institutions. The provision of well-resourced infrastructure and mission statements promoting research-informed policy and practice were advocated. Access to commissioned systematic or rapid reviews would generate research and inform policy development [19, 22–27, 30, 55–60]. External regulation included government research institutes, health licensing boards and legislation, for example, United Kingdom National Institute of Clinical Excellence [61] and the Australian Health Practitioner Regulatory Agency [62].

### Theme 3: Systems, tools, resources and time

All frameworks mentioned the importance of providing infrastructure, systems and processes to promote and support a culture of enquiry and evidence [19, 22–27, 30, 55–60]. A key organisational resource that was perceived to be enabling was clear and well documented research-related policies and procedures, including research responsibilities being explicit in all allied health job descriptions [22, 25, 27, 58, 59]. The guidelines for workforce recruitment and retention could include documenting career pathways with research components and assigning dedicated clinical research positions [12, 23, 24]. It also included allied health research being mapped in strategic plans [19, 25, 27, 59] and annual reports [19, 25, 27, 58, 59]. An in-house ethics committee or easy access to a local research ethics committee was also facilitatory [23, 24, 58, 59].

Human resources processes, such as mandatory quality and research training, were considered to be of benefit [26–28, 30, 54, 58–60]. The documentation of research outputs and research dissemination strategies, such as communities of practice, research committees, research seminars and research newsletters, were considered important [19, 23–27, 30, 54, 58–60]. Infrastructure recommendations included the routine provision of information technology services and equipment supporting research, such as 24 h intranet access, library access on line, laptops and tablets, and access to statistical and bibliographic software [19, 23–28, 30, 54, 58–60]. Collaboration between the healthcare practice settings and an academic institute was also considered highly beneficial to research capacity-building [19, 23–28, 30, 54, 58–60]. This could be formalised by joint research leadership appointments and industry research partnerships [19, 23–28, 30, 54, 58–60].

### Theme 4: Attributes of individual clinicians

There are important attributes and capabilities of individual clinicians that strengthen and develop research interactions and increase research receptivity. These include research skills and literacy, communication skills, confidence and motivation [26]. To be able to build research capacity, it is essential for the allied health workforce to be able to access, understand and apply research evidence [19, 23–28, 30, 54, 58–60].

To build research capacity it is also necessary for individual clinicians to strengthen and develop research partnerships, develop confidence and increase research receptivity [19, 23–28, 30, 54, 58–60]. Individuals need training to acquire research literacy [25, 27, 28, 30, 54, 58–60]. They can also be enabled to become research active by having ready access to mentors, research champions and multidisciplinary research collaboration networks. Practical assistance can come in the form of training in scientific writing, conference presentations, public speaking skills, journal clubs and applying for research funding [19, 23–28, 30, 54, 58–60]. Individual recognition for research achievements through awards, incentives and promotion can also assist the adoption of research-led practice [19, 23–28, 30, 55, 56].

## Discussion

This rapid review identified 16 research capacity-building frameworks that could inform the policies, principles and design of systems for the embedding of a research culture into allied health clinical practice (Table 1). The data have been synthesised to identify essential elements for embedding a culture of research within allied health clinical practice. There were two key allied health-specific models – Golenko’s Thematic Model for Research Capacity Building [25] and Hulcombe’s Health Practitioner Research Capacity and Culture Building Framework [28]. There was another that was specific to speech pathology [30] and two that were primary care and included allied health, medicine and nursing [56, 60]. The frameworks highlighted the importance of high-level systems, organisational governance and regulations that support and value allied health research. They also noted the importance of hospital leaders, and allied health managers in particular, being research literate and advocates of allied health research [25, 29].

Of value were explicit local systems and procedures for research conduct and regulation, including policies and procedures on research ethics, methods, consumer involvement, research documentation, data storage, and the dissemination of research findings to end users. Hulcombe et al. [29] stressed the importance of ensuring physical resources and time to support a research-informed workforce. The establishment of allied health research networks and formal partnerships with tertiary institutions, research institutes and industry partners also helped to embed a research culture within allied health [12, 20–22, 54–60].

Overall, the key systems factors found in this review to support allied health research were the existence of allied health research policies together with government level advocacy, support and regulations [19, 22, 25, 28, 30, 31, 54, 56, 58, 63]. Strengthening the research capabilities of individual policy-makers and assisting them and their organisations to make greater use of evidence was arguably a necessary first step in generating better evidence-informed policy [25, 27, 54, 55, 57–59, 63].

The key enabling organisational factors were leadership within organisations (especially allied health managers), collaboration, mentorship and resources. For allied health research capacity and culture to be developed and sustained, a whole-of-organisation approach was optimal [19, 25–28, 54, 56–60] and support from senior management was essential [19, 23–28, 54, 56–60, 63]. Research can be incorporated into the organisational structure, processes and core business such as strategic plans and mission statements [26–28, 54, 57–60]. Systems that establish career pathways including research-active leadership positions, research champions, conjoint university positions and research literacy were viewed as helpful [12, 19, 26–28, 30, 54–56, 58, 59, 63].

At the organisational level, collaborations between healthcare practice settings and academic institutes such as universities were perceived to have major impact [19, 26–28, 30, 54–56, 58, 59]. For change to be sustained, it was recommended that institutions provide incentives for adoption of evidence-informed behaviours [8, 39, 58, 59, 63]. An institutionalised method was preferable [17, 22, 25–28, 35, 54] and could be achieved through an external regulatory body such as demonstrated by the United Kingdom National Institute of Clinical Excellence [61].

Common to all included frameworks were the themes of strong leadership and management investment. Strengthening the capacity of individuals and organisations is necessary but probably insufficient in isolation to ensure the sustainability of evidence-informed policy-making. Strengthening of institutional capacity and regulatory control arguably requires resources, legitimacy and regulatory support from policy-makers [19, 22–28, 30, 54–60].

### Practice implications

To support the development of research capacity and capability in allied health, policy-makers and healthcare organisations can optimise capability-building frameworks, models and strategies. The identification of approaches suited to the local environment, caseload mix and workforce profile facilitates implementation. Regulation, strong leadership and supportive management structures form essential elements of a successful research culture within allied health [7, 8, 12, 19, 22–28, 30, 34, 54–60]. The future lies in new policies informed by a robustly derived framework.

### Limitations

We made every effort to source hard-to-reach publications by using forward and backward citation tracking, government websites, hand-searching and expert communication. Nevertheless, some policy documents or publications not in Web of Science or SCOPUS may have been missed. Moreover, there are more than 20 allied health professions and the literature reviewed may not have addressed issues for each. The literature reviewed predominantly focused on physiotherapy, psychology, social work, podiatry, pharmacy, occupational therapy and dietetics.

Some of the included frameworks did not demonstrate robust development methods and some were government reports. There was a paucity of evidence to support the implementation of these particular capability and capacity-building models in clinical organisations and any measures of their impact or effectiveness. Despite the method quality limitations there was, however, a consensus across all frameworks on the fundamental domains and items. The conceptual relationships between the themes are beyond the scope of this rapid review and await further investigation.

## Conclusion

This systematic review and critical evaluation of the literature identified 16 theoretical frameworks that could inform the development of models to embed a culture of allied health research into public and private healthcare services. The framework elements inform policy development, as well as the design of systems and linkages to support knowledge generation, research implementation and knowledge translation. The results will inform future allied health research capacity-building frameworks at government and policy level to oversee investment, evidence uptake and research implementation. The challenges facing policy-makers to support the use of evidence in policy cycles is considerable. Safer, more effective and efficient consumer-oriented care is the ultimate goal. Research-led and evidence-informed allied health practice also facilitates workforce recruitment, retention and capability.

## Appendix 1

### Example search strategy: Ovid MEDLINE (up until 15/10/2017)

1. Capacity Building/ or (“capacity building” or “research capacity” or (system* adj2 capacit*) or ((“research” or “allied health”) adj5 capacit*) or ((build* or increas* or develop* or enhanc* or strengthen* or *motiv*) adj5 (capacit* or skill* or abilit* or workforce)).ti,ab
2. Research/ or (culture or clin* research* or health research*).ti,ab
3. Models, Organisational/ or Models, Theory/ or Models, Theoretical/ or Systems Theory/ or (theor* or framework* or construct? or model* or concept*).ti,kw,kf. or (theor* or framework* or construct? or model* or concept* or heuristic* or lens or paradigm* or principle? or pre-engagement or phase? or stage? or “innovation support”).ab
4. Health Planning/ or Health Systems / or Health Systems Plans/ or Organisation/ or Organisational Culture/ or Organisational Innovation/ or Organisational Objectives/ or Leadership/ or Governance/ or (system or systems or systemic or “health care” or “health administration” or (translat* or implement*)) or organisation*).ti,ab
5. 1 and (2 or 3) and (3 or 4)

## Appendix 2

**Table 3:** Excluded papers and reasons for exclusion

Author,Year	Title	Reason excluded
Borkowski, 2016 [17]	Research culture in allied health: A systematic review. Aust J Primary Health. 22(4):294–303.	Not a framework; enablers and barriers
Byrne, 2014 [39]	Developing a national mentorship scheme to enhance the contribution of clinical academics to health care. Nurs Res. 22(2): 23–28.	Not a framework; enablers and barriers
Cooke, 2008 [40]	An evaluation of the ‘Designated Research Team’ approach to building research capacity in primary care. BMC Fam Pract. 9: 37.	Evaluation of framework implementation
Du Plessis, 2007 [64]	Opinions on a strategy to promote nurses’ health research contribution in South Africa. Health SA Gesondheid. 12(4):25–35.	Not a framework; Delphi study
Elphinstone, 2015 [65]	Untapped potential: Psychologists leading research in clinical practice. Aust Psych. 50(2): 115–121.	Not a framework; research capacity measurement
Friesen, 2017 [66]	Research culture and capacity in community health services: Results of a structured survey of staff. Aust J Prim Health. 23(2): 123–131.	Survey to inform enablers and barriers
Frontera, 2006 [67]	Rehabilitation Medicine Summit: Building Research Capacity: executive summary. Am J Occup Ther. 60(2):165–176.	Enablers and barriers
Gerrish, 2017 [33]	Implementing clinical academic careers in nursing: an exemplar of a large healthcare organisation in the United Kingdom. J Res Nurs. 22(3):214–225.	Framework for career development and not research capacity
Grange, 2005 [37]	Building research capacity. Nurs Manag (Harrow).12(7):32–37.	Not a framework; enablers and barriers
Holden, 2012 [27]	Evaluating a team-based approach to research capacity building using a matched-pairs study design. BMC Fam Pract.13:16.	Not a framework; an intervention study
Janssen, 2013 [68]	Building the research capacity of clinical physical therapists using a participatory action research approach. Phys Ther. 93(7): 923–34.	Not a framework; qualitative study of Physical Therapists
Joss, 2005 [69]	Workforce development to embed mental health promotion research and evaluation into organisational practice. Health Prom J Aust. 18(3): 255–259.	Not a framework
Judd, 2013 [70]	Building health promotion capacity in a primary health care workforce in the Northern Territory: some lessons from practice. Health Prom J Aust. 24(3):163–169.	Not a framework; practitioner survey
Misso, 2016 [20]	Development, implementation and evaluation of a clinical research engagement and leadership capacity building program in a large Australian health care service. BMC Med Educ. 16: 13.	Not a framework; Protocol for an intervention study
Moore, 2015 [71]	Council for allied health professions research: Collaborative initiative to develop and promote research capacity and influence. Physiother. 101, eS1027-eS1028.	Conference abstract; no data
Pickstone, 2008 [29]	Building research capacity in the allied health professions. Evidence Policy. 4(1): 53–68.	Not a framework
Probst, 2015 [36]	Research from therapeutic radiographers: An audit of research capacity within the UK. Radiography. 21(2):112–118.	Not a framework; survey and audit
Segrott, 2006 [32]	Challenges and strategies in developing nursing research capacity: a review of the literature. Internat J Nurs Stud. 43(5): 637–651.	Not a framework; enablers and barriers
Skinner, 2015 [35]	Embedding research culture and productivity in hospital physiotherapy departments: Challenges and opportunities. Aust Health Rev. 39(3):312–314.	Framework for translation; not research capacity-building
Varshney, 2016 [38]	Understanding collaboration in a multi-national research capacity-building partnership: a qualitative study. Health Res Pol Syst.14: 1–10.	Qualitative study; enablers and barriers
Wenke, 2016 [12]	The role and impact of research positions within health care settings in allied health: a systematic review. BMC Health Serv Res. 16: 355.	Not a framework
Wenke, 2017 [14]	Allied health research positions: a qualitative evaluation of their impact. Health Res Pol Syst. 15(1):6.	Not a framework
Williams, 2015 [72]	Research capacity and culture of the Victorian public health allied health workforce is influenced by key research support staff and location. Aust Health Rev. 39(3):303–311.	Not a framework; investigation of enablers and barriers

## Abbreviations

CASP: Critical Appraisal Skills Programme
MMAT: Mixed Methods Appraisal Tool
PRISMA: Preferred Reporting Items for Systematic Reviews and Meta-Analyses
